# Extracting Potential New Targets for Treatment of Adenoid Cystic Carcinoma using Bioinformatic Methods

**DOI:** 10.61186/ibj.27.5.294

**Published:** 2023-03-27

**Authors:** Tayebeh Forooghi Pordanjani, Bahareh Dabirmanesh, Peyman Choopanian, Mehdi Mirzaie, Saleh Mohebbi, Khosro Khajeh

**Affiliations:** 1 Department of Biochemistry, Faculty of Biological Science, Tarbiat Modares University, Tehran, Iran;; 2 Department of Applied Mathematics, Faculty of Mathematical Sciences, Tarbiat Modares University, Tehran, Iran;; 3 ENT and Head & Neck Research Center, the Five Senses Health Institute, Rasoul Akram Hospital, Iran University of Medical Sciences, Tehran, Iran

**Keywords:** Adenoid cystic carcinoma, Adipogenesis, Biomarkers, IGF type 1 receptor

## Abstract

**Background:**

Adenoid cystic carcinoma is a slow-growing malignancy that most often occurs in the salivary glands. Currently, no FDA-approved therapeutic target or diagnostic biomarker has been identified for this cancer. The aim of this study was to find new therapeutic and diagnostic targets using bioinformatics methods.

**Methods:**

We extracted the gene expression information from two GEO datasets (including GSE59701 and GSE88804). DEGs between ACC and normal samples were extracted using R software. The biochemical pathways involved in ACC were obtained by using the Enrichr database. PPI network was drawn by STRING, and important genes were extracted by Cytoscape. Real-time PCR and immunohistochemistry were used for biomarker verification.

**Results:**

After analyzing the PPI network, 20 hub genes were introduced to have potential as diagnostic and therapeutic targets. Among these genes, *PLCG1* was presented as new biomarker in ACC. Furthermore, by studying the function of the hub genes in the enriched biochemical pathways, we found that IGF-1R/IR and PPARG pathways most likely play a critical role in tumorigenesis and drug resistance in ACC and have a high potential for selection as therapeutic targets in future studies.

**Conclusion:**

In this study, we achieved the recognition of the pathways involving in ACC pathogenesis and also found potential targets for treatment and diagnosis of ACC. Further experimental studies are required to confirm the results of this study.

## INTRODUCTION

About 40 types of salivary glands malignancy have been identified and distinguished by histology. ACC is the most prevalent neoplasm of the salivary glands after mucoepidermoid carcinoma and is currently diagnosed by histological analysis of a biopsy or surgical sample. Differential diagnosis is made between ACC and other benign or malignant neoplasms in the same areas^[^^1^^]^. Despite tumor growth are well-controlled using surgery and radiotherapy, ACCs often have a poor long-term prognosis^[^^2^^]^. More than 40% of ACC cases show distant metastasis in which lung, bone, and liver are the most common sites of metastasis^[^^3^^]^. Furthermore, ACC tends to spread along the craniofacial nerve trunk, making this tumor very destructive and unpredictable^[^^4^^]^. Since no chemotherapy is available for patients with unresectable tumors^[^^5^^]^, it is imperative to identify novel and effective biomarkers involving in tumorigenesis and drug resistance of ACC. To better understand the biochemical pathways contributed to ACC pathogenesis, studying the signaling pathways using bioinformatics tools can be helpful. Molecular studies of ACC have been performed to determine the genomic sequence of patients and the expression profile of mRNAs involved in the disease.

Studies have demonstrated that most ACC cases contain a translocation between chromosomes 6 and 9, which connects *MYB* to *NFIB* transcription factor locus or other enhancers and creates different fusion with *MYB*, followed by *MYB* overexpression^[^^1^^,^^6^^]^. MYB protein contributes to regulating the transcription of many genes, including those involving in the RNA processing, cell cycle, and DNA repair, thereby promoting tumor growth^[^^7^^]^. Targeting transcription factors is complicated, and there is still no drug to target MYB^[^^8^^]^; hence, it is necessary to identify applicable targets for ACC treatment by understanding the mechanism of tumorigenesis. Although most ACC tumors show high *MYB* expression, it cannot be used as a biomarker to diagnose the disease, because some specimens show negative or poor staining^[^^9^^]^. *MYB* overexpression is also not specific to ACC and found in other tumors such as squamous cell carcinoma, which is confused with ACC^[^^4^^]^. Therefore, the aim of the present study was to unravel the dysregulated signaling pathways in ACC using bioinformatics and computational analysis to extract potential therapeutic and diagnostic targets. One of the valuable tools for this goal is the analysis of data obtained from cDNA microarray with PPI network and enrichment analysis.

## MATERIALS AND METHODS

**Screening DEGs**

We searched ACC in the GEO database (https://www.ncbi.nlm.nih.gov/geo/) and then selected the “expression profile by array” option. Two datasets, GSE59701 and GSE88804, were considered for the present analysis. The dataset GSE59701 (submission year, 2015; year of last update, 2018) contains 12 ACC along with 12 normal samples^[^^10^^]^. The dataset GSE88804 (submission date, 2016; last update, 2018) comprises of 13 ACC and 7 normal samples^[^^11^^]^. The raw data of the mRNA expression profiles were downloaded as MINiML files. DEGs between ACC and normal samples in each dataset were extracted separately using the limma package in R software (version 3.6.0; https://www.r-project.org//). |logFC|>1 and adjusted p < 0.05 were set as the cut-off point, which means the results are statistically significant^[^^12^^-^^14^^]^. After extracting DEGs, the upregulated and downregulated genes in the two datasets, GSE59701 and GSE88804, were collected and used for the subsequent analysis. 

**GO and pathway enrichment analyses of DEGs**

Enrichr (http://amp.pharm.mssm.edu/Enrichr) is a comprehensive web-based tool for gene set enrichment analysis^[^^15^^]^. GO analysis in the categories of molecular function, biological process, and cellular component was performed using Enrichr. In addition, the KEGG pathway enrichment of DEGs was conducted to identify the signaling pathways of the involved DEGs. First, we obtained the overlapping DEGs by Venn diagram (https://bioinformatics.psb.ugent.be/webtools/Venn/). Then we used 363 overlapping downregulated genes from two studies to find the downregulated pathways and used 397 overlapping upregulated genes to discover the upregulated pathways from KEGG. Adjusted p < 0.05 was considered the cut-off criteria of statistical significance. We analyzed the enriched pathways, based on DEGs, to find out which axes in each pathway are dysregulated in the ACC samples compared to the normal. Afterwards, we established the connection between the axes based on the KEGG data. Correlation between dysregulated pairs of KEGG pathways was obtained and then demonstrated using a heatmap.

**PPI network construction and hub gene exploration**

A total of 761 DEGs from the upregulated and downregulated pathways, identified by Venn diagram, were employed to generate a PPI network using STRING database (https://string-db.org)^[^^16^^]^. After uploading genes into the STRING website, the organism was set to Homosapiens, and the minimum required interaction score was adjusted to the medium confidence(0.4). PPI network data were exported in tab-separated values format and imported to Cytoscape software version 3.8.0 for visualization and analysis of the molecular interaction networks^[^^17^^]^. CytoHubba, a plugin tool in Cytoscape^[^^18^^]^, was applied to identify hub genes according to three topological analysis methods, including edge percolated component, maximum neighborhood component, and degree, and one centrality method, named betweenness. To evaluate the diagnostic power of hub genes, a ROC curve was generated using the pROC package in R software^[^^19^^]^. By calculating the AUC for 20 hub genes, five genes with the highest AUC were plotted in a ROC curve. 

**Correlation between the expression of hub genes and the** ***MYB*** **oncogene**

In order to investigate the relationship between the expression of 20 hub genes and *MYB* oncogene, we calculated the correlation coefficient of the hub gene expression with *MYB* and plotted the correlation coefficient in a heatmap. 

**Real-time PCR**

Seven pairs of tumor and tumor margins (as normal specimens) from seven ACC patients were obtained from the Tumor Bank of Amir Alam Hospital, Otorhinolaryngology Research Center, Tehran University of Medical Sciences, Tehran, Iran. RNA extraction from the samples was performed using TrizoLEX (DNA Biotech; cat no: DB9683; Tehran, Iran). The extracted RNA was observed on 1.5% agarose gel to ensure its quality. We obtained the values of A260/280 and A260/230 to determine the RNA contamination and concentration using a nanodrop 2000 spectrophotometer (Thermo Fisher Scientific, USA). After ensuring the quality of RNA extraction, cDNA synthesis was performed using a cDNA Synthesis Kit (cat no: YT4500, Yekta Tajhiz, Iran). Specific primers were designed using AlleleID software and included GAPDH-F: ATTCTCTGATTTGGTCGTATTGGG, GAPDH-R: ATGACAAGCTTCCCGTTCTC, PLCG1- F: AGTCACATTGCTTTGTCATTCTCT, and PLCG1-R: GCTGATATACGATCCTCACGATTC. The relative gene expression for *PLCG1* gene was obtained with the RealQ Plus Master Mix Green (Ampliqon, Denmark) using Applied Biosystems StepOne™ thermal cycler. *GAPDH* was selected as the endogenous control. After obtaining the Ct values, LinRegPCR software was used to evaluate the primer efficiency. Fold changes were obtained using the E^-ΔΔCt^ method. To draw the bar plot, the value of gene expression in the normal samples was taken as one, and the fold change in tumor tissues was measured. 

**Immunohistochemistry of** **PLCG1**

Three formalin-fixed, paraffin-embedded blocks of tissue from three ACC patients were collected. The margins of tumor site were considered as normal tissue to compare PLCG1 expression. The tissue sections were prepared from the samples. The slides were placed in TBS 1× solution (T5912; Sigma, USA) inside the microwave, which was turned off after reaching the boiling point, and the samples remained in the solution for 20 minutes. Samples were then washed with PBS (in three steps at 5-min intervals). H_2_O_2_ and methanol were mixed in a ratio of 1 to 9 and placed on the samples for 10 minutes. The samples were washed again with PBS, and the primary antibody (cat. no orb333981; Biorbyt, UK) diluted with PBS (1 to 100) was poured onto the samples and placed at room temperature for one hour. The samples were then rinsed three times with PBS, each time for 5 minutes, and 100 µl of Linker (PVP1000D; Diagnostic BioSystems, USA) was added to the samples for 15 minutes. Following washing, polymer solution (100 µl; Diagnostic BioSystems) was added to the sample for 30 minutes. The samples were washed again with PBS, and then 100 µl of DAB solution (ACV999; ScyTek, USA) was added to the sample. After five minutes, the samples were washed with water and finally placed in hematoxylin dye for 10 seconds. After washing, dehydration and clarification steps, and photography was carried out using a light microscope (Labomed, USA) by a pathologist. Data were analyzed by IMAGE J software version 1.48. Adjusted p < 0.05 was considered as cut-off criteria of statistical significance.

**Statistical analysis**

All data analyses were presented by mean ± SEM. Significant differences groups were determined by multiple comparisons using One-way ANOVA, followed by Tukey’s post hoc test. p < 0.05 was used as the criteria for statistical significance.

## RESULTS AND DISCUSSION

**Fig. 1:**
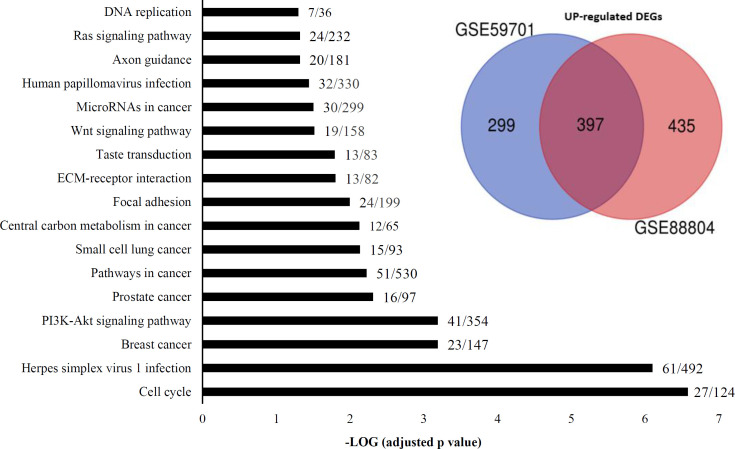
**KEGG pathway enrichment analyses of** **the** **upregulated DEGs in ACC samples vs. normal samples.** **Seventeen pathways have an adjusted** **p** **< 0.05. The vertical axis represents the names of the pathways. The horizontal axis represents the statistical significance calculated based on the adjusted p value. The numbers on the graphs indicate the number of genes changed in a pathway, which is divided by the total number of genes in that pathway**

Two gene expression microarray datasets, GSE59701 and GSE88804, were obtained from GEO. Using the limma package in R software. A total of 2,190 DEGs, including 1,131 upregulated and 1,059 downregulated genes, were obtained from two expression profile data. In addition, 760 overlapped DEGs, including 397 upregulated and 363 downregulated genes, were retrieved from the two datasets using Venn diagram. The GO analysis and KEGG signaling pathway enrichment of the 2,190 DEGs were performed using the Enrichr database. We considered adjusted p < 0.05 as the threshold to get meaningful pathways. Seventeen statistically significant pathways were also upregulated (Fig. 1). PI3K-Akt, cell cycle, central carbon metabolism in cancer, focal adhesion, extracellular matrix-receptor interaction, Wnt, axon guidance, mRNAs in cancer, and Ras are important pathways involved in ACC obtained from the pathway enrichment analysis using Enrichr. 

**Fig. 2:**
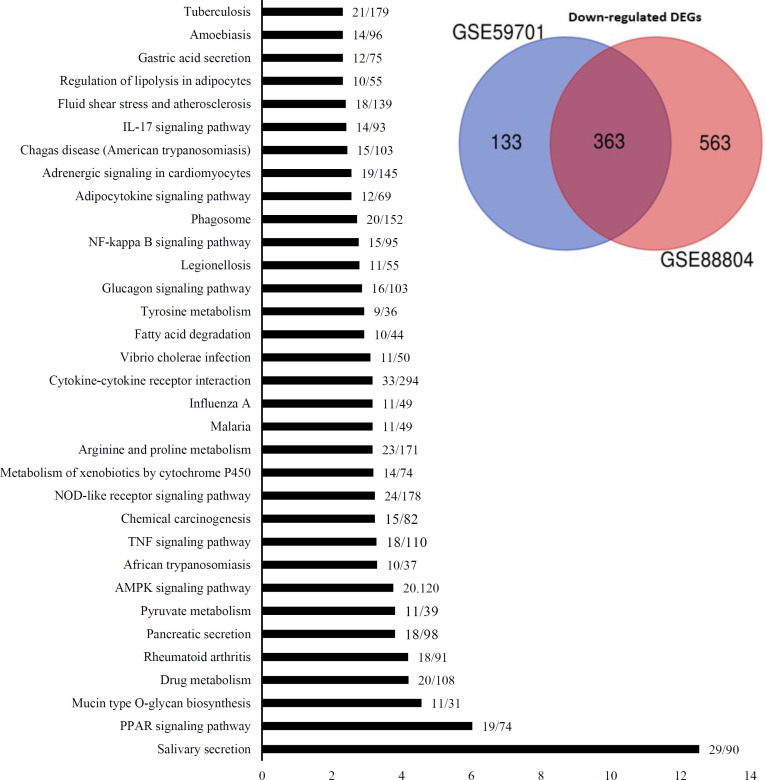
**KEGG pathway enrichment analyses of** **the** **downregulated DEGs in ACC vs. normal samples.** **All the 33** **pathways have an adjusted p** **< 0.05. The vertical axis represents the names of the pathways. The horizontal axis represents the statistical significance calculated based on adjusted p value. The numbers on the graphs indicate the number of genes changed in a pathway that is divided by the total number of genes in that pathway**

**Fig. 3:**
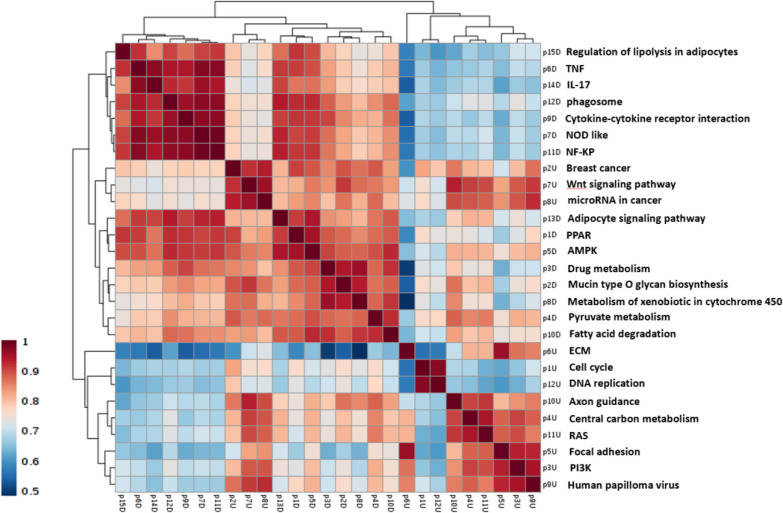
**The h** **eatmap representing the correlation between the dysregulated pairs of KEGG pathways involved in ACC.** **The color range from red to blue indicates high correlation between two pathways to low correlation**

**Fig. 4:**
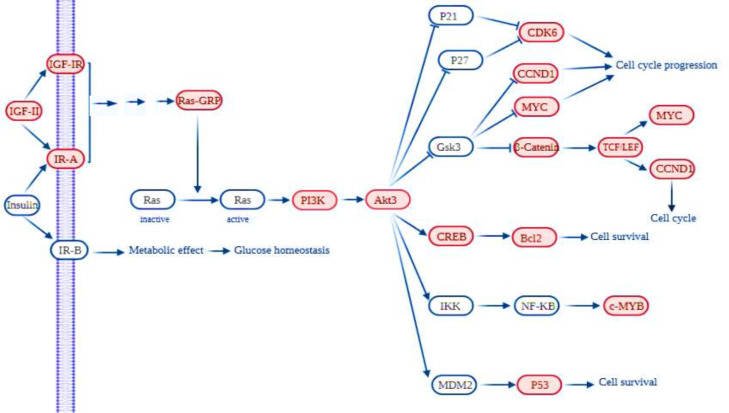
**A simple schematic of the connection between the upregulated pathways in ACC, including IGF-1R/IR, RAS, PI3K/Akt, and Wnt based on KEGG pathways. Red** **capsule** **indicates overexpressed genes according to DEGs**

Results exhibited 33 downregulated pathways, with the adjusted p < 0.05 (Fig. 2), which can be divided into three groups. One group is related to salivary secretion. The second group is associated with the lipid metabolism and adipocyte differentiation, including the peroxisome proliferator-activated receptor, AMPK, and adipocytokine signaling pathways. The third group related to immune response and inflammation that includes rheumatoid arthritis, tumor necrosis factor, nucleotide oligomerization domain-like receptor, NF-kappa B, IL-17, and phagosome signaling pathways. As indicated in Figure 3, the members of each group have a high expression correlation with each other. Analysis of the significant enriched pathways, including PI3K-Akt, Ras, Wnt, and cell cycle identified by Enrichr shed more light on the procedure of tumorigenesis of ACC. Figure 4 shows the upregulated axis in ACC, which was drawn from the integration of pathways mentioned above. 

**Fig. 5:**
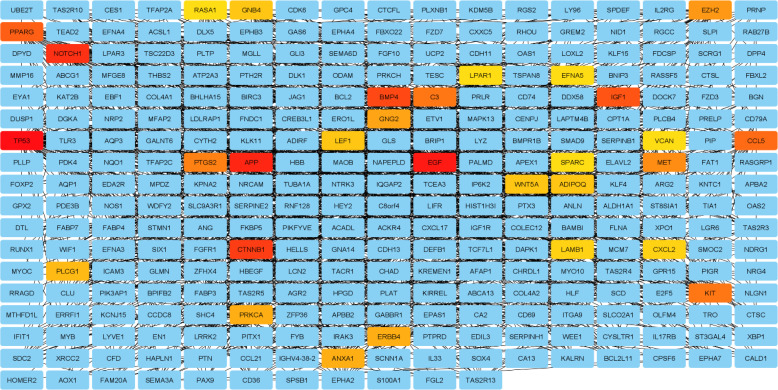
Protein-protein interaction network of overlapping DEGs between two datasets. Thirty genes with the highest degree are shown in red to yellow color and others are shown in blue

**Fig. 6:**
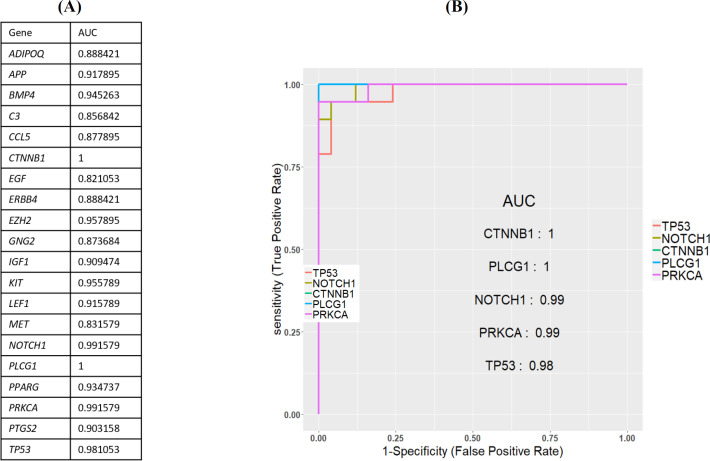
**(** **A** **) AUC calculation for 20 hub genes** **;** **(** **B** **) ROC curve of five genes with the highest AUC**

**Fig. 7:**
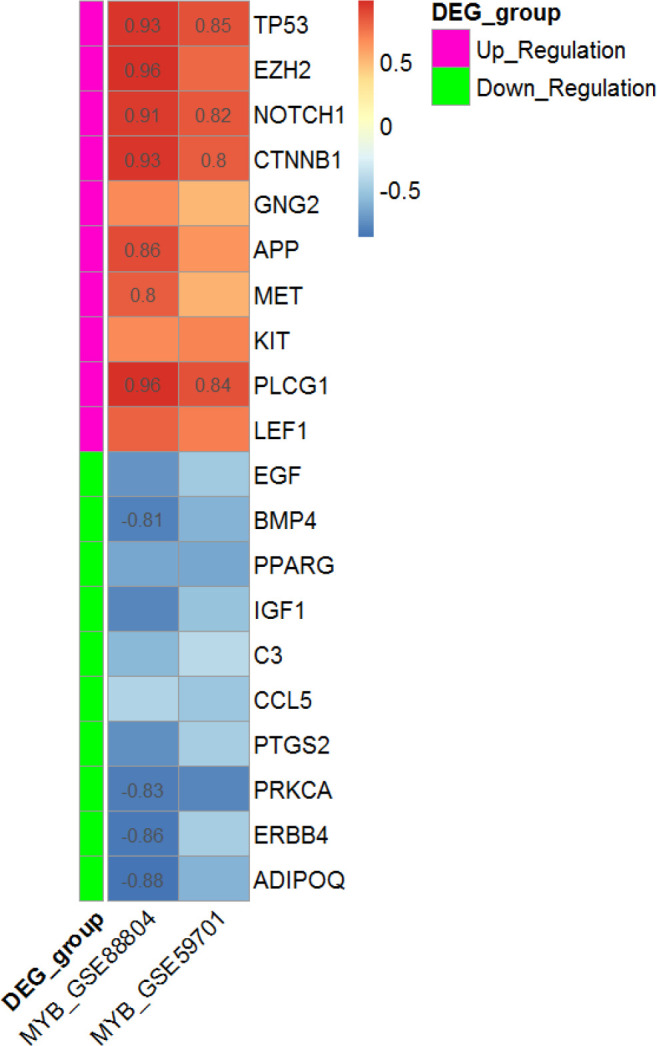
The heatmap showing the correlation between the expressions of 20 hub genes with MYB oncogene in two separate datasets. The color ranges from red to blue indicates positive to negative correlation

**Fig. 8:**
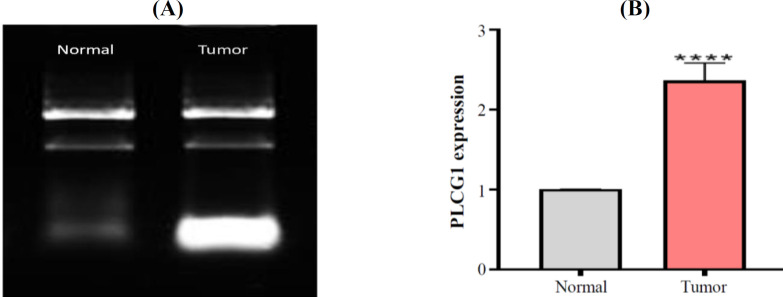
**(A) Gel electrophoreses of mRNA extraction from normal and tumor tissues; (B)** ***PLCG1*** **expression in tumor compared to the normal tissues using the real-time PCR (** ^****^ **p = 0.0006)**

**Fig. 9:**
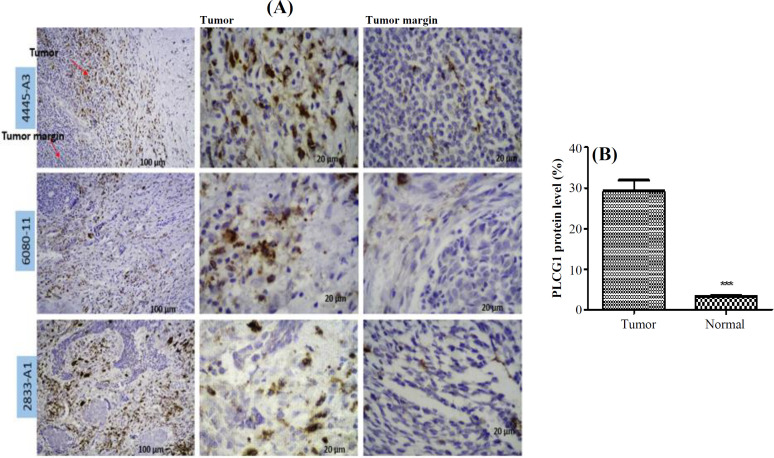
(A) Examining the expression of PLCG1 in tumor and tumor margin cells of three ACC samples. Arrows show the names of three samples. (B) Bar plot of amount of PLCG1 in tumor cells relative to tumor margin considered as normal (^****^p = 0.0001)

The PPI network of 760 overlapped DEGs was constructed using the STRING database and Cytoscape software (Fig. 5). The hub genes were obtained using four methods, including edge percolated component, maximum neighborhood component, degree and betweenness separately. In Figure 5, 30 genes with the highest degree are shown. Of the 30 genes, 20 were also confirmed by three other methods, including edge percolated component, maximun neighborhood component, and betweenness. *TP53*, *EZH2*, *NOTCH1*, *CTNNB1*, *GNG2*, *APP*, *MET*, *KIT*, *PLCG1*, and *LEF1* were considered as the upregulated and *BMP4*, *PPARG*, *IGF1*, *C3*, *CCL5*, *COX2*, *PRKCA*, *ERBB4*, *ADIPOQ*, and *EGF* as downregulated hub genes. The substantial participation of some of these 20 hub genes, such as *TP53*^[^^20^^]^, *NOTCH1*^[^^21^^]^, *CTNNB1*^[^^22^^]^, *MET*^[^^5^^]^, and *KIT*^[^^23^^]^, in the ACC tumorigenesis has already been studied. The importance of other hub genes in ACC has not been studied and need further research. Calculation of AUC for 20 hub genes was performed to validate their potential as diagnostic biomarkers (Fig 6A). *CTNNB1*, *NOTCH1*, *PLCG1*, *PRKCA*, and *TP53* genes have an AUC of more than 0.98, indicating their high specificity and sensitivity to distinguish the ACC samples from normal ones. Figure 6B demonstrates the ROC curve for the five above-mentioned genes. To investigate relationship between the expression of hub genes and *MYB* oncogene, the correlation of 20 hub gene expression with *MYB* expression was calculated and shown by a heatmap (Fig. 7). We found that in addition to *TP53*, *CTNNB1*, and *NOTCH1*, which have a decisive role in ACC^[^^20^^,^^24^^,^^25^^]^, expression of *PLCG1* is highly correlated with *MYB* expression. Investigation of the *PLCG1* expression using real-time PCR showed an increase of more than two times in the tumor relative to normal tissues (p < 0.0001; Fig. 8). IHC staining with anti-PLCG1 antibody also revealed that *PLCG1* expression increased about 10 times in the tumor tissues compared to the normal ones (Fig. 9). This observation, together with the results obtained from the ROC curve, proposes this gene as a new diagnostic or therapeutic biomarker for future studies in ACC. In this line, a considerable role has been reported for *PLCG1* in some cancers. In a study conducted on breast cancer, the high expression of phosphorylated *PLCG1* predicts metastasis in patients undergoing adjuvant chemo-therapy^[^^26^^]^. In another study, PLCG1 inhibition induced programmed cell death in lung adenocarcinoma A549 cells^[^^27^^]^. 

*EZH2* is a hub gene that appears to be important, particularly in tumorigenesis and involves in the histone methylation and inhibition of some tumor suppressors. The expression product of this gene is found in only active dividing cells^[^^28^^]^; therefore, it can be used as a diagnostic marker for these cells^[^^29^^]^. EZH2 can interact with Wnt signaling factors such as *c-myc* oncogene and cyclin D1^[^^30^^]^. Given that some available US food and Drug Administration-approved EZH2 inhibitors are used for treating different cancers^[^^31^^]^, investigation on EZH2 in ACC can be of great importance. The signaling pathways obtained herein can also provide new information about the mechanism of ACC tumorigenesis. Figure 4 shows the role of IGF-2, along with the IGF-IR, in drug resistance in ACC. 

Genomic sequencing data and cytogenetic maps have revealed that the majority of ACC cases had translocations, leading to the juxtaposition of *NFIB*, *TGFBR3*, and *RAD51B* super-enhancers either in the upstream or downstream of *MYB* locus. MYB transcription factor binds to these translocated super-enhancers and makes a looped structure containing the *MYB * promoter and increases its expression^[^^11^^,^^32^^]^. Increased MYB transcriptional regulatory activity promotes tumor cell proliferation in ACC, highlighting MYB as a potential therapeutic target^[^^4^^]^. Interestingly, Andersson et al.^[^^2^^,^^5^^]^ found that MYB-NFIB expression was regulated by inhibiting the IGF1R pathway; however, IGF-IR/IR inhibition had a short-term clinical response, and the patient became resistant to treatment after a few months. The reason for this drug resistance was the interaction of the IGF-1R pathway with other signaling pathways^[^^33^^]^. The IGF system has two ligands, IGF-1 and IGF-2, and three receptor, IGF-1R (primarily), IGF-2R, and the IR, which in turn IR has two variants named IR-B and IR-A^[^^34^^]^. According to our DEG analysis, the three above-mentioned receptors were upregulated, but IGF-1 was downregulated in ACC. Also, analysis of the upregulated pathways obtained from KEGG showed the pivotal role of PI3K-AKT and RAS signaling pathways in the tumorigenesis of ACC (Fig. 4). These pathways are activated with IGF-IR or IR^[^^35^^]^. Mitogen signaling by IR has been described in some tumor models, and several studies have been performed, in which the IGF1R and IR compensate the inhibition of each other^[^^34^^]^. Evidence has disclosed that many cancer cell types, including prostate, colorectal, breast, and lung cancers, express not only the IGF1R but also the IR-A, an isoform with high affinity for both insulin and IGF-2 and is associated with a poor prognosis^[^^36^^]^. By activating IR-A, IGF-IR, and IGF-1R/IR-A hybrid, IGF-2 can function as a part of the drug resistance development system against IGF-1R inhibitors^[^^34^^,^^37^^-^^39^^]^. A solution to overcome this problem is to directly target the IGF-2 ligands because IGF-2 inhibitors, in addition to having antiproliferative activity, do not interfere with IR-B function and glucose metabolism^[^^40^^]^. Overall, IGF-2 could be a valuable new therapeutic target for ACC that has not yet been studied in ACC patients and requires future experiments. 

PPARG is the second pathway that can be targeted in ACC treatment. Based on the signaling pathway enrichment, the pathogenesis of ACC is mainly linked to lipid metabolism, in which the related signaling pathways, including adipocyte, PPARG, and AMPK, are downregulated. Figure 3 shows a high correlation among the pathways involving in the lipid metabolism. Interestingly, there was a link between lipid metabolism and the IGF-1R pathway. IGF-1 promotes preadipocyte proliferation and differentiation, but IGF-IR abundance increases with adipocyte dedifferentiation^[^^41^^]^. IGF-2 has an inhibitory effect on the differentiation of visceral adipocytes confirmed by reducing the expression of PPARG and ADIPOQ, two differentiation markers of adipocytes. Visceral adipocyte plays a substantial role in the pathogenicity of various diseases such as metabolic syndrome, type 2 diabetes, and cardiovascular risk^[^^41^^]^. IR-A is the predominant isoform in visceral preadipocytes and makes them more responsive to IGF-2. IR-B predominates in the subcutaneous preadipocytes; hence, the binding of insulin to these cells regulates glucose homeostasis. Many types of tumors (breast, gastric, renal, colon, and ovarian tumors) grow in the proximity of visceral adipocytes and induce dedifferentiation of visceral adipocytes into pre-adipocytes or reprogram them into cancer-associated adipocytes. Dedifferentiation of adipocytes causes the release of fatty acids into tumor microenvironment and supports the tumor growth^[^^42^^]^. If differentiation of these preadipocytes is induced again, the process of carcinogenesis may be prevented^[^^43^^]^. Herein, we observed that PPARG pathway is strictly inhibited in ACC samples rather than normal samples (Fig. 2).

We also found *PPARG * and *ADIPOQ* as hub genes in the PPI network. PPARG belongs to the nuclear hormone receptor superfamily named PPARs. An earlier study has suggested a significant reduction in PPARG expression in follicular thyroid, esophageal, cervical, and colon cancers^[^^44^^]^. Activation of the PPARG pathway with its agonists may prevent tumor growth and proliferation by inhibiting PI3K and Ras, the downstream pathways of the insulin/IGF axis^[^^45^^]^. After activation of PPARG, it moves to the nucleus and binds to DNA to regulate the transcription of several genes, which ultimately increases the storage of fatty acids in adipocytes and differentiation of adipocytes^[^^46^^,^^47^^]^. It has been displayed that Ciglitazone, a synthetic PPARG ligand, prevents the proliferation of A549 (human alveolar adenocarcinoma) cells^[^^44^^]^. Furthermore, PPARG activation by rosiglitazone and pioglitazone substantially induces apoptosis and cell cycle G2 arrest in bladder cancer cells^[^^48^^]^. Although the connection between the PPARG and IGF pathways has not clearly been recognized, the therapeutic function of PPARG is observed in tumors in which IGF pathway is upregulated^[^^49^^]^. In light of these pieces of evidence, PPARG agonists may be considered as potentially preventive and therapeutic agents in ACC. In support of this hypothesis, there is a report indicating that metformin usage significantly improves disease-free survival in ACC patients^[^^50^^]^. The use of these drugs completes the effect of tyrosine kinase inhibitors in ACC treatment. Interestingly, using metformin in A549 cells reduced PLCG1 levels and induced autophagy^[^^51^^]^; hence, there is a need for further research to uncover the effect of PPARG-activating drugs in the treatment of ACC. 

The third group of pathways that decreased with a high correlation in the ACC (Fig. 3), was the pathways relating to inflammation and the immune system, including tumor necrosis factor, NF-kappa B, nucleotide oligomerization domain-like receptor, rheumatoid arthritis, adipocytokine, IL-17, and phagosome signaling pathways. While the progression and invasion of cancer cells are mediated by proinflammatory factors in the tumor microenvironment, tumor-derived factors sometimes disrupt the host immune system, leading to anti-inflammatory conditions in the tumor micro-environment. This immunosuppressive situation is associated with tumor progression and poor prognosis for patients with advanced cancer^[^^52^^]^. Identifying the mechanism of immune system suppression in the ACC and finding the role of immunosuppressive factors derived from tumor cells in disease progression, provide new insights into ACC treatment through the host immune system activation. These results lead to new perspective on drug target proteins in ACC for experimental biologists in the future. 

A recent study has identified 20 hub genes in ACC using bioinformatics methods^[^^53^^]^. In that study, 41 samples from three mRNA expression profiles (GSE36820, GSE59702, and GSE88804) were analyzed. In the present study, 44 samples were chosen from two mRNA expression profiles of GEO database (GSE59701 and GSE88804). In order to obtain more accurate data, we tried to carefully select samples from the GEO database; for instance, not adding xenograft samples to tissue samples due to the importance of homogeneity, we deleted xenograft samples from tissue samples. Overall, the selection of different samples and certain methods used herein for extracting hub genes, compared to a recent bioinformatics study^[^^53^^]^, led to the acquisition of 20 different hub genes. Also, in the same study, DEGs were enriched in SOX2, AR, SMAD, and MAPK signaling pathways, which are different from our study. Considering that the DEG extraction method is similar in both investigations, the discrepancy in the results of these two studies is probably due to the difference in the selection of samples. On the other hand, kinase enrichment analyses showed the importance of IR and IGF-IR expression in tumorigeneses of ACC^[^^53^^]^, which are in accordance with our results. 

 In this research, we extracted two new therapeutic targets for ACC treatment using bioinformatics tools and based on previous investigations. Dysregulation of IGF-IR/PI3K/Akt axis in ACC due to the increase of IGF-2 plays a crucial role in tumorigenesis. Thus, inhibition of IGF-2 instead of IGF-IR/IR is suggested to avoid resistance to treatment and interference with glucose metabolism. Furthermore, inhibition of adipogenesis causes the release of fatty acids from adipocytes into the tumor microenvironment, which helps tumor growth. Hence, activation of the PPARG pathway can reduce the available sources for tumor cells by differentiating adipocytes. Besides, from PPI network analysis of DEGs, we identified 20 hub genes, including *TP53*, *EZH2*, *NOTCH1*, *CTNNB1*, *GNG2*, *APP*, *MET*, *KIT*, *PLCG1*, *LEF1*, *BMP4*, *PPARG*, *ADIPOQ*, *IGF1*, *COX2*, *C3*, *CCL5*, *PRKCA*, *ERBB4*, and *EGF*. Among them, PLCG1 has an essential role in tumorigenesis of breast cancer^[^^26^^]^ and lung adenocarcinoma^[^^27^^]^ and its role in ACC has not been studied. *PLCG1* expression has a high correlation coefficient with *MYB*, as well as the highest AUC score in the ROC curve. We observed a significant increase in the expression of *PLCG1* in tumor cells compared to the tumor margin*. *Due to the rarity of ACC, we were able to obtain a limited number of samples for experimental results*. *Further experimental studies are definitely required to confirm the results of the present study.

## DECLARATIONS

### Acknowledgments

The authors acknowledge the financial support of this study by Tarbiat Modares University and Iran University of Medical Sciences, Tehran, IRan. We also thank Otorhinolaryngology Research Center of Amir Alam Hospital for providing ACC samples for this study. 

### Ethical statement

The study sampling protocols were approved by Iran University of Medical Sciences, Tehran, Iran.

### Data availability

The datasets analyzed during the current study are available in the GEO database (https://www.ncbi.nlm. nih.gov/geo) with GSE59701 and GSE88804 accession numbers. 

### Author contributions

TFP: investigated and interpreted of data, and wrote the article; BD: revised the article and helped interpret the data; PC: collected and analyzed data; MM: designed the methodology; SM: evaluated research goals and aims and reviewed and edited the manuscript; KK: supervised the study and reviewed and edited the manuscript. All authors have read and approved the final version of the manuscript.

### Conflict of interest

None declared.

### Funding/support

We are grateful to Tarbiat Modares University and Iran University of Medical Sciences, Tehran, Iran for financial support of this research.
